# Unusual presentation of a neuroepithelial cyst: A case report

**DOI:** 10.1016/j.radcr.2022.08.047

**Published:** 2022-09-17

**Authors:** Fallis Desita, Widiana Ferriastuti, Dyah Fauziah

**Affiliations:** a Department of Radiology, Faculty of Medicine Universitas Airlangga, Dr Soetomo General Academic Hospital, Surabaya, Indonesia; b Department of Anatomic Pathology, Faculty of Medicine Universitas Airlangga, Dr Soetomo General Academic Hospital, Surabaya, Indonesia

**Keywords:** Neuroepithelial cysts, Brain cyst, MRI

## Abstract

Neuroepithelial cysts are benign, congenital, ependymal-lined cysts that constitute an infrequent cause of neurological symptoms among intracranial cystic lesions. We present Indonesian patient who underwent brain MRI depicting neuroepithelial cysts and pathological examination confirmed. Surgery was performed and yielded a satisfying result. Awareness of the entity and specific imaging features is key to making the diagnosis of neuroepithelial cyst and commencing appropriate treatment. This report is expected to aid in the diagnosis of neuroepithelial cysts, which are rare, and reduce the occurrence of misdiagnosis.

## Introduction

Neuroepithelial cysts are cavities with an ependymal or epithelial lining. They contain fluids of unidentified origin and develop in the central nervous system. Regarding ventricular or subarachnoid spaces, there is no clear communication between them. Moreover, the cysts are sporadic or evolve congenitally in the central nervous system and represent less than 0.4% of all cyst-like lesions. Those indications are commonly found in East Java [1], [2], [3]. The cavitations emerge mainly in intracranial locations, such as intraparenchymal, intraventricular, and subarachnoid spaces; the spinal cord is an unusual site of occurrence [1,4].

The profound literature has described this mass as an ependymal cyst, neuroepithelial cyst, glioependymal cyst, and neuroglial cyst. These descriptors indicate the complexity of this disease's terminology [5,6]. We illustrate a case of neuroglial cyst with accompanying visual and movement disturbances.

## Case description

A 29-year-old male patient presented with blurred vision for the past 2 years. He complained of weakness on the left side of the body, slurred speech, and a skewed face 1 year prior to hospitalization. The symptoms worsened in severity over time and persisted during the course. Upon admission, he was unable to walk and had lost the vision in his left eye. No account of head trauma or oncologic records was reported.

**Fig. 1:**
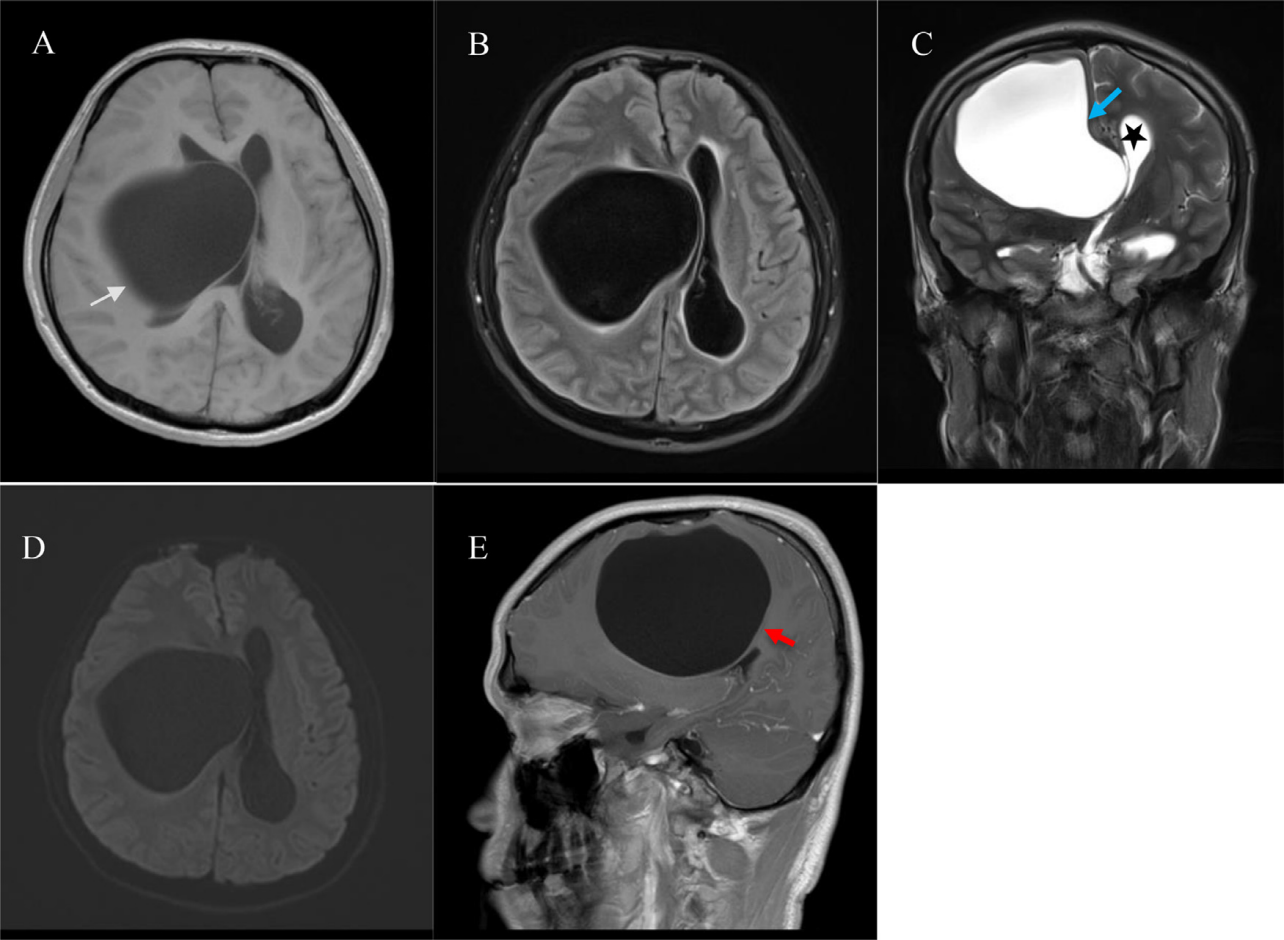
T1-weighted axial (A) plane and FLAIR (B) image showed the cyst with a well-defined thin wall in low signal (arrows). T2-weighted coronal (C) plane showed high signal intensity (blue arrows), compressing the interventricular foramen responsible for hydrocephalus (star). (D) Diffusion-weighted imaging (DWI) showed unrestricted lesions. T1-weighted sagittal plane (E) with contrast media injection showed no enhancement (red arrows).

Neurological examination revealed left-sided motor weakness without sensory disturbances, increased left-sided deep tendon reflexes with pathological reflexes, and normal cerebellar functions. Moreover, on ophthalmological examination, the light perception in the left eye was remarkably decreased. Fundoscopy detected right eye papilledema and left optic papilla atrophy. MRI examination of the head revealed a large, well-demarcated, intraparenchymal cystic lesion measuring 8.7 × 7.8 × 7.8 cm in the right frontoparietal lobe. It produced a significant mass effect on the surrounding parenchyma and resulted in noncommunicating hydrocephalus (Fig. 1). The characteristics of the cystic fluid component were similar to CSF in all sequences without contrast enhancement following intravenous gadolinium administration. Regarding the proximity to the subarachnoid space and ventricle, there was displacement of the right and left ACA. Furthermore, the mass surpassed the left side and the right MCA to the superior side. No blood vessels were related to the cyst.

**Fig. 2:**
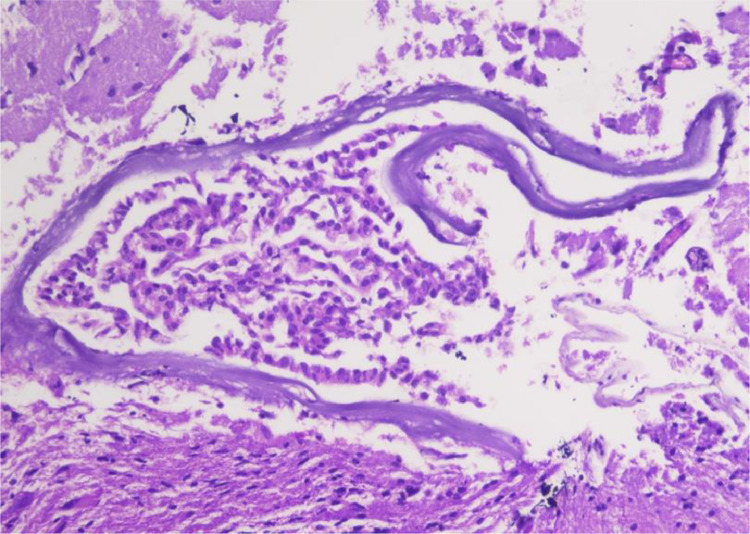
Histology of the wall from the neuroepithelial cyst, showing brain tissue with visible edges of the cyst wall layered with the cuboid and columnar cells with a rounded core, fine chromatin, inundated with the cytoplasm. No signs of malignancy were evident. Magnification ×200.

A mini craniotomy was conducted in the left frontal lobe to aspirate the cysts with insertion of an Ommaya reservoir. The cysts contained clear fluid without signs of recent or old hemorrhage. Histopathological examination was requested for the surgery afterward (Fig. 2). The procedure yielded an excellent outcome, with improved neurological status. As a result, the patient was discharged from the hospital after 2 weeks.

## Discussion

Neuroepithelial cysts, also known as glioependymal cysts, originate from the area surrounding the craniospinal axis (ie, orbital, intraspinal, perispinal, sacral, and pericranial). The cysts develop from ectopic ependymal cells [4]. These congenital cysts exist in the remnants of the entrapped residues of an embryonic neural tube inside the growing white matter. This localization correlates with the occurrence of the cysts age of twenties to thirties, with no sex predilection. Intracranial neuroepithelial cysts are categorized as sporadic compared to the more common arachnoid cysts. They are considered benign in nature and are slow-growing lesions that commonly arise in an intracranial intracerebral location. They are most frequently found in a supratentorial location (74.1%), with a predilection for the frontal lobe [4,^7^,8]. Moreover, clinical manifestations vary in presentation (ie, seizures, motor deficit, headache, nausea, parkinsonism, visual symptoms, and coma).

In contrast, the infratentorial GEC produces more unusual symptoms, such as hemifacial spasm, dizziness, and diplopia [1,4]. The origin of such lesions is under research, and it is postulated that they arise from ectopic ependymal cells during the embryonic period; the ectopic cells develop into an either intra- or extra-axial epithelial-lined cyst [1].

Both MRI and CT play an essential role in establishing a diagnosis, although MRI is the preferred modality for identifying neuroepithelial cysts. The cyst has smooth, rounded borders and is composed of thin, unilocular walls that contain CSF-like fluid that is identifiable on CT or MRI. CT scans generate a cystic focus with unenhanced low-density fluid, and MRI shows a cystic-filled lesion with fluid that is hypointense on T1-weighted and hyperintense on T2-weighted images. Moreover, the contents of the cyst show no restriction in the diffusion-weighted sequence, which is comparable to CSF and no surrounding edema. The mass effect caused by these cysts is minimal, even in prominent lesions, indicating their marginal growth, especially in adults. There is no enhancement after contrast media injection [1,^4^,^9^,10].

In this case, three differential diagnoses were reported: porencephalic, arachnoid, and epidermoid cysts. Porencephalic cysts circulate inside the white matter, replicate the CSF, and exert no mass effect on the neighboring tissue. Moreover, there is no enhancement with contrast administration that connects them to the ventricular system or subarachnoid space with adjacent gliosis. Arachnoid cysts exist as a singular, extra-axial cyst with a well-demarcated margin and share similar intensity to CSF. They dislodge the surrounding parenchyma, dent the skull bone, and exhibit no restriction on DWI sequence and are unenhanced on contrast administration. For this reason, most arachnoid cysts originate from above the tentorium and have no ventricular attachments. Epidermoid cysts contain a lobular cavity filled with CSF-like substance and exhibit gradual expansion features. Subsequently, they produce mass effect, invading the surrounding anatomy and wrapping nearby nerves and vessels. As demonstrated in fluid-attenuated inversion recovery (FLAIR) sequences, an epidermoid cyst covers the area of the heterogeneous regions of hyperintensity. In contrast, the emergence of CSF and hyperintensity on diffusion-weighted imaging sequence matters. Occasionally, there is calcification (10%-25%) and moderate peripheral enhancement [9], [10], [11].

These cavitating lesions are usually filled with clear, CSF-like fluid; however, several reports have indicated a variety of intracavitary contents that appeared xanthochromic, opalescent, milky, or turbid. Pathological examination reveals a partially ciliated cuboidal or columnar lining epithelium lying on the astroglial tissue bundles, interceded by the basal membrane. Surgical intervention is indicated based on the dependable location of the cyst and the subarachnoid or ventricular space approximation. The emergence of simple puncture and open or stereotactic drainage has resulted in a higher cyst recollection rate [10].

Several reports and literature discuss the radiological characteristics and management of such entities, investigating their sporadic occurrence and other benign, clinical, or subclinical intracranial cystic lesions. We observed one rare case of neuroepithelial cysts with clinical manifestations of visual and movement disorders. The findings of our study focused on the diagnosis of clinical and imaging features with histological evidence. Furthermore, we emphasized the algorithmic approach, based on the most expected anatomical sites, signs, symptoms, and radiological evidence. These approaches take into consideration the features of the lesions to narrow the differential diagnosis of intracranial cysts. Therefore, a systematic approach is key to evidence-based management of incidental benign variants (ie, neuroglial cysts) and avoiding unnecessary surgical interventions.

## Conclusion

Neuroglial cysts are congenital central nervous system lesions with challenging preoperative detection as this disorder correlates with many clinical and radiological characteristics of other anomalies. Nevertheless, histopathological studies remain the only method of producing a definitive diagnosis. The symptoms depend on the cyst's location and size; however, some patients may present with mild symptoms in cases of large lesions. In this case, surgery was the preferred modality for the symptomatic patient, and the location of the cyst determined the type of surgery, which also took into consideration the ventricular or subarachnoid spaces that needed to be approximated. Therefore, radiology has an essential role in diagnostics and may aid physicians with their diagnosis and surgical approach.

## Patient consent

Informed consent was obtained for publication of a case report. Written informed consent was obtained from the patient for the publication of this case report.
